# Supplementary material for: Tuning of liver circadian transcriptome rhythms by thyroid hormone state in male mice
Source: Sci Rep. 2024 Jan 5;14:640. doi: 10.1038/s41598-023-50374-z (PMC10770409; doi:10.1038/s41598-023-50374-z)
Supplement: Supplementary file 6 — Supplementary Information 6. [file 41598_2023_50374_MOESM6_ESM.docx]

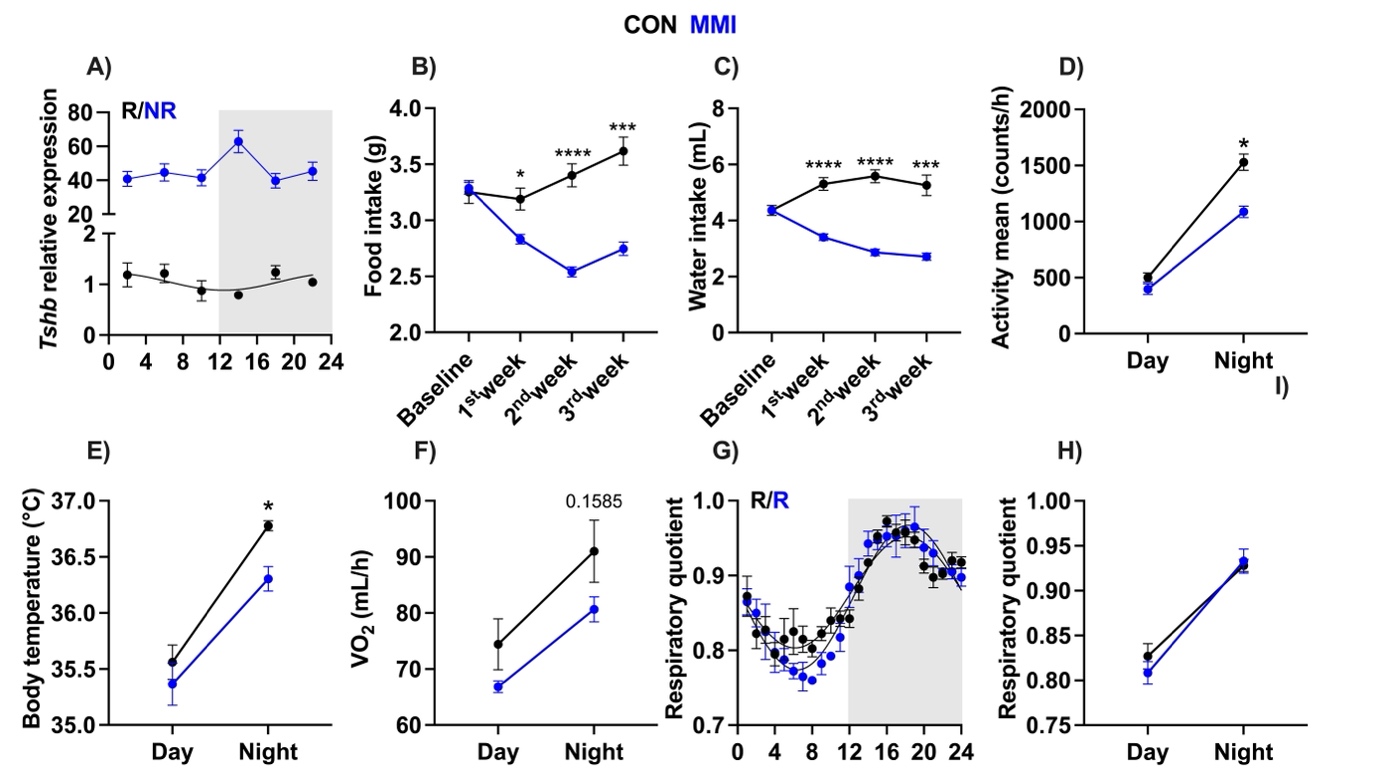


**Supplementary Figure 1: Evaluation of the systemic metabolic parameters of the CON and MMI mice.** A) Diurnal *Tshb* mRNA expression in the pituitary. B – C) Assessment of food and water intake. D– F) Metabolic parameters (described in the y-axis) were obtained from the 3rd week of the experiment. Day and night data were plotted by averaging values from ZT 0 to 12 (day) and from ZT 12 to 24 (night). Asterisks indicate significant differences between the CON and MMI mice. G – H) 24-hour profiles of respiratory quotient and day and night comparisons. In A, n = 3 - 4. In B and C, n = 8 (per cage). In D, n = 4 and 5 for the CON and MMI groups, respectively. In E – H, n = 4 for each group. *, ***, and **** represent p values of < 0.05, 0.001, and 0.0001, respectively.

**
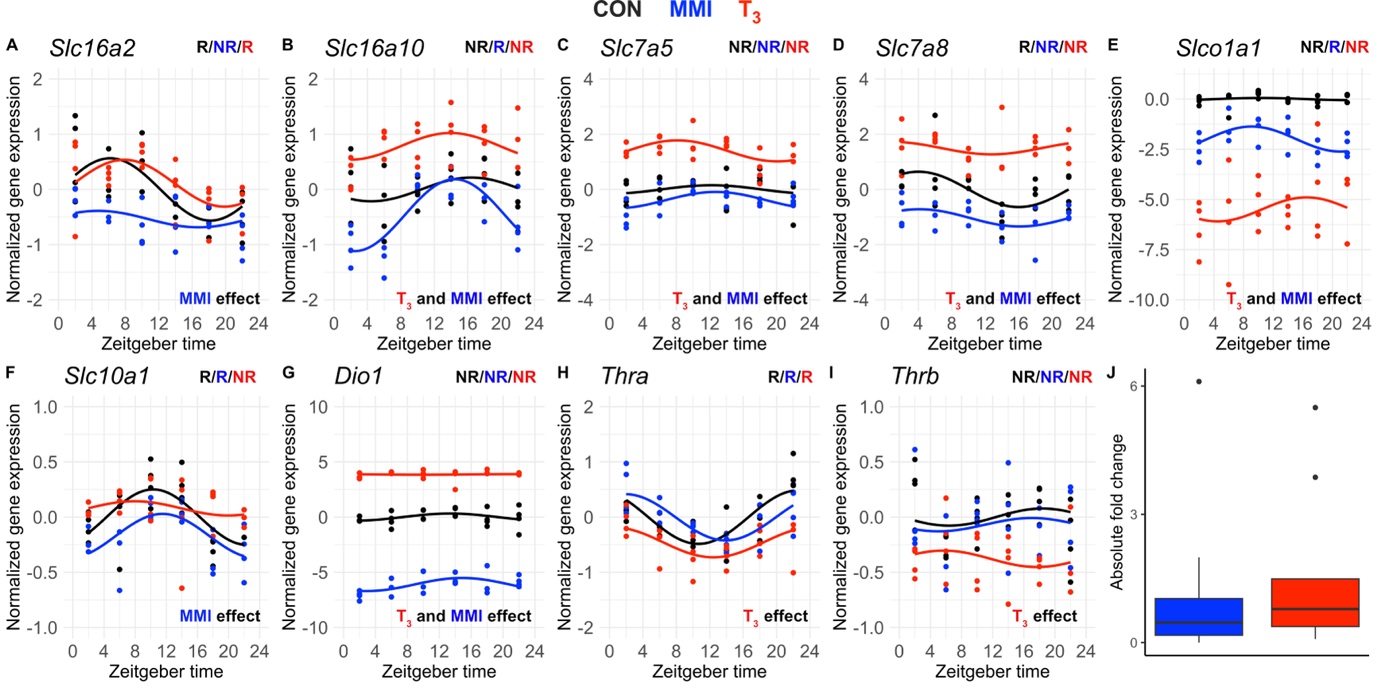
**

**Supplementary figure 2: Expression of thyroid hormone regulator genes is affected by low and high TH state.** A – I) Diurnal expression profiles of selected classical TH regulator genes are shown. Presence (R) or absence of significant circadian rhythmicity (NR) by JTK cycle (p value < 0.05) is depicted. J) Average absolute fold change comparisons of all TH regulators (n = 9) are shown (non-significant). Effects of low- or high- TH states were estimated using two-way ANOVA (main treatment effects, (p < 0.05). n = 3 – 4 for all ZTs and groups.


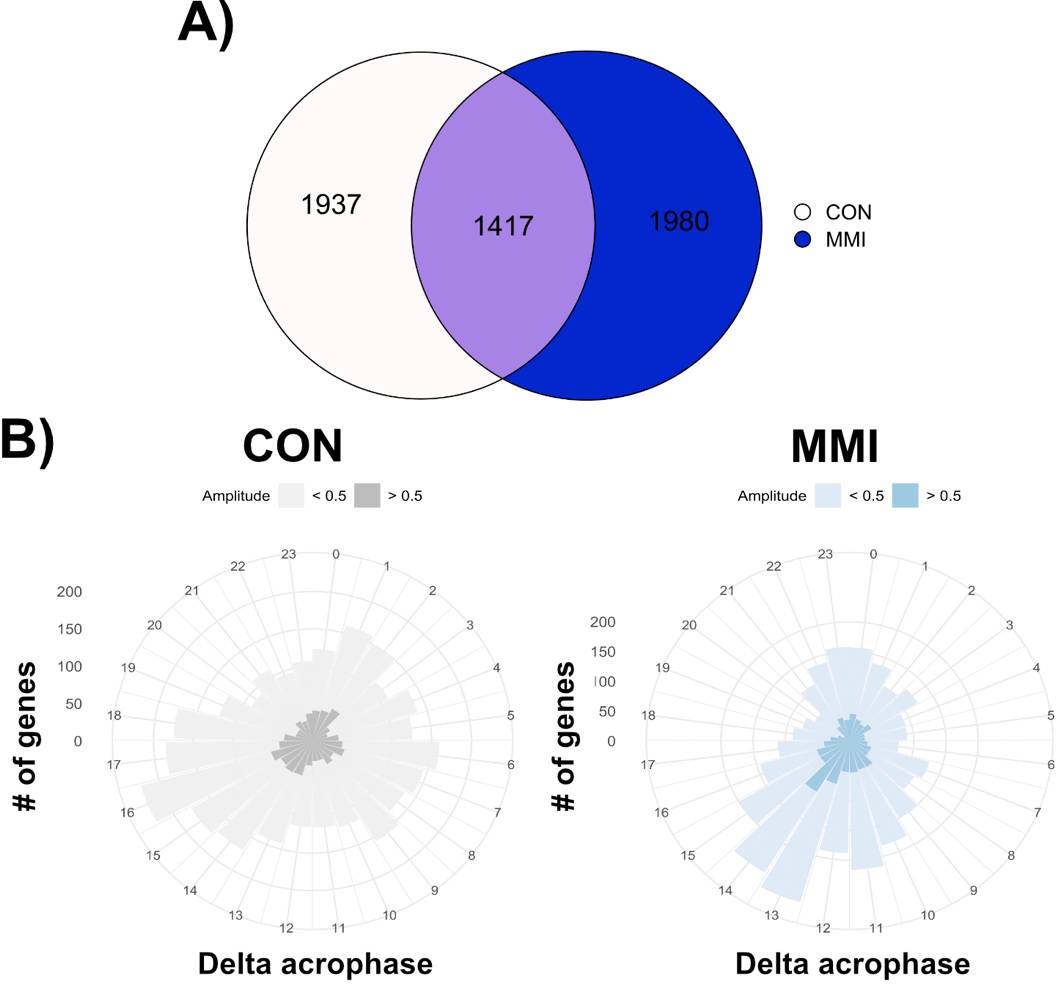


**Supplementary figure 3: Liver diurnal transcriptome characterization in low TH conditions.** A) Venn diagram showing significantly rhythmic probes identified in CON and MMI groups (JTK cycle, p < 0.05). B) Rose plots (depicting peak phase) of rhythmic genes in CON and MMI are shown.

**
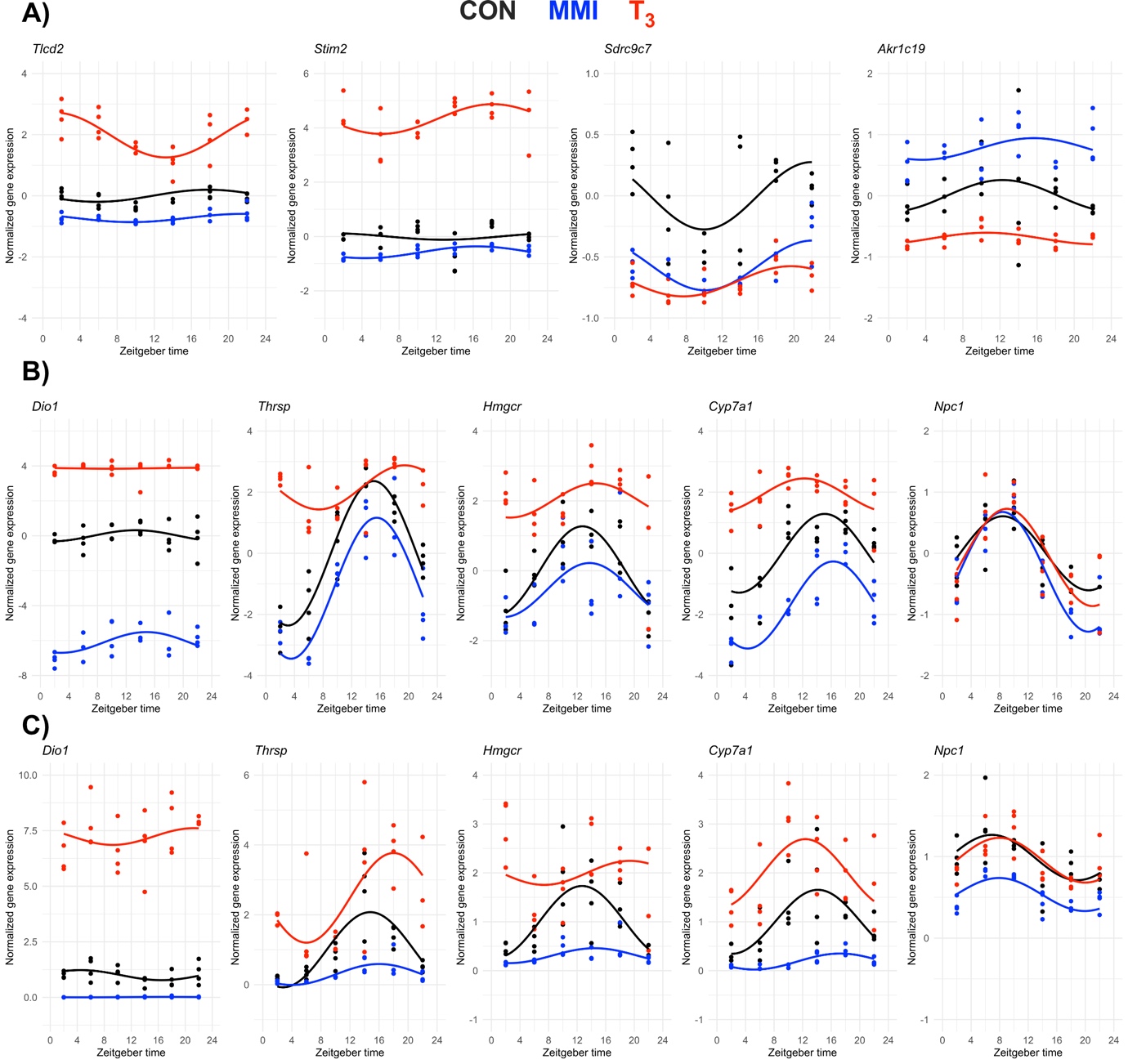
**

**Supplementary Figure 4:** Selected microarray gene validation by qPCR. A) Validation of four representative tuning genes by qPCR is depicted. B) Selected key metabolic genes identified in the microarray analyses are shown. C) Validation of the genes identified in B by qPCR is shown. n = 3 – 4 for all ZTs and the groups.
